# “Who Doesn’t?”—The Impact of Descriptive Norms on Corruption

**DOI:** 10.1371/journal.pone.0131830

**Published:** 2015-06-29

**Authors:** Nils C. Köbis, Jan-Willem van Prooijen, Francesca Righetti, Paul A. M. Van Lange

**Affiliations:** Department of Social and Organizational Psychology, VU University Amsterdam, Amsterdam, Netherlands; Defence Science & Technology Organisation, AUSTRALIA

## Abstract

Corruption poses one of the major societal challenges of our time. Considerable advances have been made in understanding corruption on a macro level, yet the psychological antecedents of corrupt behavior remain largely unknown. In order to explain why some people engage in corruption while others do not, we explored the impact of descriptive social norms on corrupt behavior by using a novel behavioral measure of corruption. We conducted three studies to test whether perceived descriptive norms of corruption (i.e. the belief about the prevalence of corruption in a specific context) influence corrupt behavior. The results indicated that descriptive norms highly correlate with corrupt behavior—both when measured before (Study 1) or after (Study 2) the behavioral measure of corruption. Finally, we adopted an experimental design to investigate the causal effect of descriptive norms on corruption (Study 3). Corrupt behavior in the corruption game significantly drops when participants receive short anti-corruption descriptive norm primes prior to the game. These findings indicate that perceived descriptive norms can impact corrupt behavior and, possibly, could offer an explanation for inter-personal and inter-cultural variation in corrupt behavior in the real world. We discuss implications of these findings and draw avenues for future research.

## Introduction

Imagine the following situation: you work as a CEO of a construction company, which competes for an enormous bridge building contract. The Ministry of Public Affairs allocates this contract to the company with the best tender. Yet, instead of going down the legal path and trying to out-compete the other companies, you discover another way to attain the contract: the responsible Minister has a soft spot for Paris and would love to go on a private vacation. You realize that using some of your company’s budget to invite the Minister to a vacation might be money well spent. Such an invitation will ensure you an advantage in the bridge building project while putting the other competitors in a disadvantaged position. Would you do it?

This example portrays a form of corruption–defined in this context as “misuse of an organizational position or authority for personal or organizational (or sub-unit) gain, where misuse in turn refers to departures from accepted societal norms”[1]. Corruption generally disrupts the functioning of groups, organizations, and societies [2]. Empirical corruption research highlights various detrimental societal effects of corruption, including impaired state development [3], degraded national wealth [4], and over-exploitation of natural resources [5,6]. Corruption has elicited considerable research from various fields [7]. On the macro level multiple correlates of corruption have been identified, ranging from lack of transparency [8], over colonial history [9], to extractive institutions [10]–to name a few (see for a more thorough overview, [2,11,12]). So far, corruption research has devoted much less attention to psychological factors that help to explain why corruption is rampant in some contexts while being almost non-existent in other contexts (see for some meaningful exceptions, [13,14–16])

With regards to impactful psychological factors of corruption, political scientists [17] and economists [18] alike emphasize the importance of (perceived) social norms–the commonly held beliefs about the behavior of others [19]. As we will outline, descriptive norms help corrupt agents to estimate the likelihood of success of corrupt deals and serve as a decision-making benchmark. Given this importance, we explored the impact of descriptive norms on the decision to engage in corruption using a behavioral measure of corruption in three empirical studies.

In order to understand the way norms influence corrupt behavior we have to differentiate between two main types of norms: descriptive and injunctive norms. Descriptive norms convey information about how most people behave in a given situation. They describe the perceived *frequency* of a specific act. Injunctive norms convey information about the particular acts that most people approve or disapprove of–hence, whether this specific behavior is appropriate and/or ethical [20,21].

In the present contribution, we focus on the impact of descriptive norms on corrupt behavior for two main reasons. First, descriptive norms are subject to more inter-societal variance. That is, descriptive norms about corruption vary considerably within a given societal context [22–24]–people hold diverging beliefs about the frequency of corruption [11]. Yet, injunctive norms about corruption vary less strongly within the same societal context. People largely hold converging beliefs about corruption being generally unethical and wrong–even in contexts in which corruption is rampant [25,26]. This moral condemnation is also reflected in the law: corruption marks a crime according to most national codes of law [27] and international conventions [28].

Second, injunctive norms are less malleable than descriptive norms [17]. While the aforementioned views about corruption being wrong and inappropriate are relatively stable, the beliefs about the descriptive norms about corruption can be subject to change. Especially, in domains in which people do not have own experience with corruption, the beliefs about the frequency of corruption are malleable. In fact, changing descriptive norms is suggested as one of the most promising ways to fight corruption [17].

In sum, descriptive norms about corruption might vary substantially across and within societies, and might be malleable. In some societal contexts corruption is perceived to be ubiquitous, in other contexts it is perceived to be almost non-existent ([29], for a game theoretic model of this distinction see, [30]). Groups, organizations and societies can rest in a high corruption equilibrium or a low corruption equilibrium depending on the frequency and the *perceived* frequency of corruption in a given context. Importantly, such equilibria are not always stable [29]. In fact, a system can move from a state of high descriptive corruption norms to low descriptive corruption norms and vice versa [31]. For such a change to occur, the *belief* about the frequency of corruption is theorized to crucially impact corrupt *behavior* [17,29,32].

Think for example of bribing a police officer after having violated a traffic rule. If you believe that this type of corruption is widespread, initiating a bribe payment (e.g. by slipping a note into your driver’s license) has a high prospect of success and might help you to avoid a hefty fine. In this context, the expected value of the police officer accepting the bribe outweighs the potential punishment. However, if you believe that this form of corruption hardly ever occurs, such a practice might get you into bigger trouble than you were in the first place. In this second scenario, the potential punishment for attempting a bribe outweighs the expected value of bribe acceptance. In line with this example, it is frequently argued that the variance of corrupt behavior largely depends on whether people think others are corrupt as well and not on whether it is generally inacceptable or illegal [32–34].

Taken together, these arguments indicate that descriptive norms crucially influence corrupt behavior. Previous research has not yet tested this link experimentally, partly due to a lack of suitable methodology to assess corrupt behavior. Hence, besides making a novel contribution to corruption research by examining the impact of descriptive norms on corrupt behavior, the present study also introduces a novel corruption game. In this game, we place participants in the position of a CEO of a construction company and let them decide whether to bribe the official who allocates a bridge building contract (more details below). It resembles a frequently occurring corruption situation in the real world yet one about which the vast majority of the participants should have no first-hand experience. Hence, behavior will be more strongly impacted by perceived descriptive norms and less based on the participants’ own experience.

By embedding these bribe transactions in an economic game framework, we mask the corrupt act as invitations of a public official to different events that bring about business advantages. So instead of being explicitly asked to pay a bribe or bluntly paying money to the official, participants can engage in more subtle types of bribery. Masking corruption in that way helps to increase the variance of corrupt behavior in the game as it reduces the impact of social desirability (i.e. people not engaging in corruption because it is socially unacceptable). However, this behavior was nonetheless perceived as ‘corrupt’ as participants across all three studies perceived the invitations of the public official as significantly more corrupt than not inviting the public official (all *ps* < .016).

In this work, we set out to test whether descriptive norms–the belief about the frequency of corruption in a given context–predict corrupt behavior. Using the novel corruption game, we conducted three studies. First, we tested with two correlational studies whether the perceived descriptive norms before (Study 1) and after (Study 2) the corruption game correlate with corrupt behavior in the game. Second, in order to test the causal relationship we set up an experiment in which information about descriptive norms was manipulated to tests its impact on the subsequent corrupt behavior (Study 3).

## Study 1

### Materials and Methods

In the first study, we investigated whether perceived descriptive norms of corruption correlate with corrupt behavior. For that purpose, we assessed the perceived frequency of this specific corrupt behavior with one item prior to the corruption game (described in more detail below).

#### Participants and ethics statement

Students from the VU University Amsterdam (*N* = 66, *M*_*age*_ = 26.79, *SD*_*age*_ = 15.49; 51.5% = female) took part in the study in exchange for course credit or money (2€). Participants first answered several items assessing the perceived norms about work place related behavior–one item assessing the specific corrupt practice modelled in the ensuing corruption game. All studies reported in this contribution utilize the same basic experimental setup. Our faculty’s ethical review board (VCWE) approved of this experimental setup. In all of the studies reported in this manuscript, prior to completing any scales, participants signed a written informed consent form. Upon completion of the study, participants were debriefed and thanked for their participation. In all reported studies, prior to debriefing we assessed age, gender and education level of the participants. These demographic factors had no statistical significant effects on the corrupt decision in any of the reported studies (all *ps* > .122).

### Study design and tasks

#### A priori norms

The Work Place Norm scale [35] assesses work related behavior with 5 items (α = .724; e.g. “Copy a company owned software for your own use”). Participants indicated the perceived frequency of the described behavior on a 100-point slider answer scale ranging from ‘0’ (= nobody does it) to ‘100’ (= everybody does it). Higher scores reflect a higher perceived frequency of the respective behavior. Since none of the existing items assessed the specific corrupt behavior in the game and due to the context specificity of corruption [7], we formulated one new item to assess the norms specifically related to the corrupt behavior in the corruption game. This item states “Invite a public official for a private vacation on the company’s expenses to ensure business advantages”.

#### Corruption game

**Fig 1:**
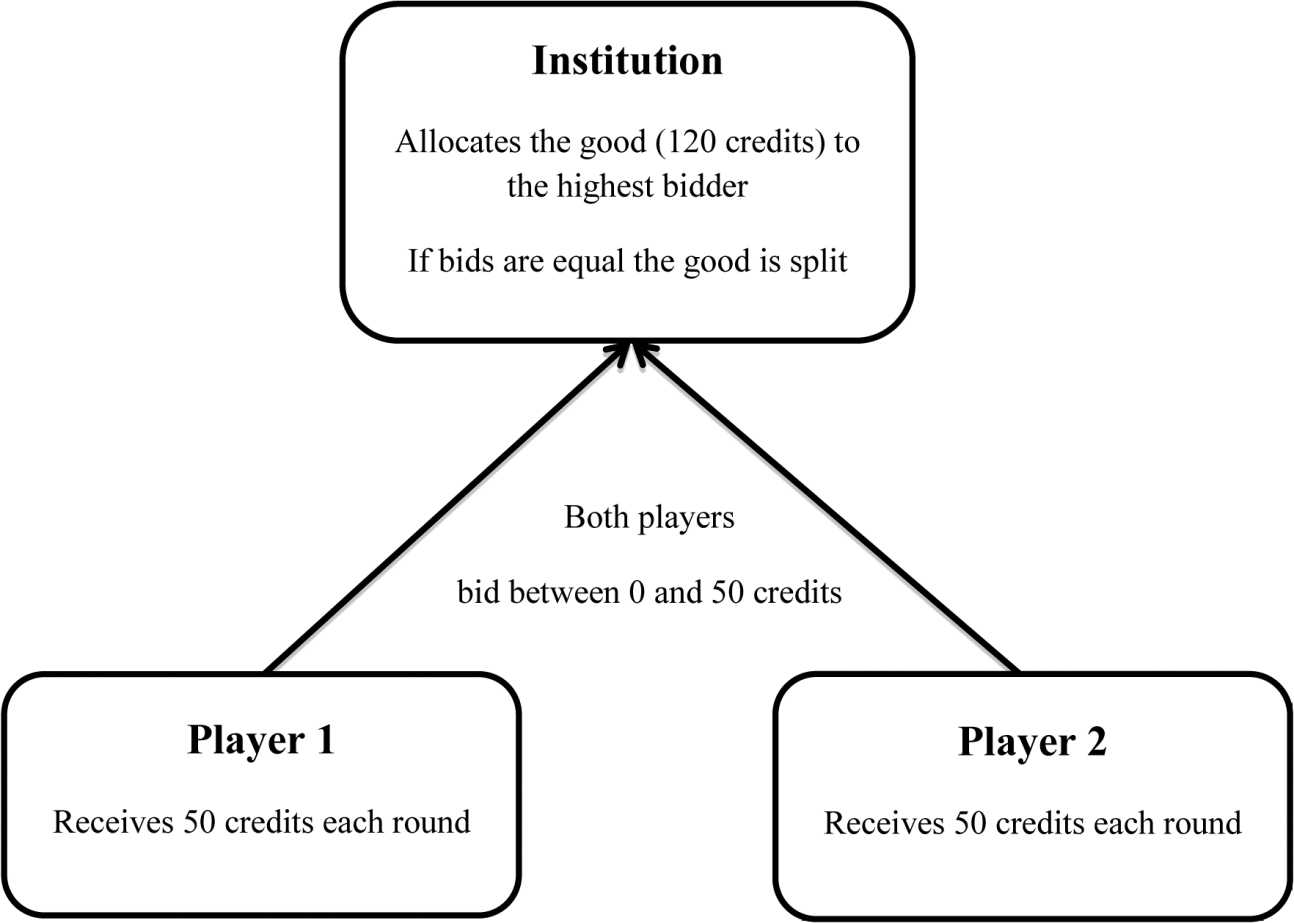
Triadic structure of the corruption game. Participants take the role of the potentially corrupt player.

The corruption game entails three players. In an auction fashion, two players compete for a total prize of 120 credits. The third player administers the prize to the highest bidder (see Fig 1). Each round both competing players receive a budget of 50 credits to make bids. The competing players can choose from an array of options, which range from not bidding at all (0 credits) to bidding their entire budget for the round (50 credits). The competing players keep the credits that they do not allocate in a bid. While the highest bidder wins the total prize, in case of both players offering the same bid, the prize is split equally between the two. The bidding process lasts for five rounds.

The payoff matrix (see Table 1) depicts all possible outcomes of this bidding process. Allocating 50 credits in the bid is the dominant strategy of this bidding process–this option results in a strict Nash equilibrium [36]. Put differently, for each player bidding 50 credits yields the best outcomes independent of the choice of the other player. We include a corrupt option for one player in this fair bidding structure. Our approach resembles the triadic structure typical for many corrupt transactions in procurement situations: two (or more) competing players–one potentially corrupt player and one fair player (i.e. a potential victim of corruption)–and a third player who resembles an official allocating the price to the highest bidder. We place all participants throughout all the experiments presented here only in the role of the potentially corrupt player.

**Table 1:** Outcome matrix of the fair bidding game.

	Player 2												
		*50*		*40*		*30*		*20*		*10*		*0*	
	***50***	**60**	60	**120**	10	**120**	20	**120**	30	**120**	40	**120**	50
	***40***	**10**	120	**70**	70	**130**	20	**130**	30	**130**	40	**130**	50
**Player 1**	***30***	**20**	120	**20**	130	**80**	80	**140**	30	**140**	40	**140**	50
	***20***	**30**	120	**30**	130	**30**	140	**90**	90	**150**	40	**150**	50
	***10***	**40**	120	**40**	130	**40**	140	**40**	150	**100**	100	**160**	50
	***0***	**50**	120	**50**	130	**50**	140	**50**	150	**50**	160	**50**	50

*Note*. The matrix illustrates the outcomes for each player before the corrupt option is introduced to the game. The range of bidding options for each player are in italics. The outcomes for player 1 are in bold. The dominant strategy for both players is allocating 50 credits.

The participants can offer a bribe to the official in order to circumvent splitting the price with the other competing player and thus ‘breaking’ the equilibrium into their favor. The game is set up so that the other player does not have the chance to bribe the Minister. As participants were informed each round which player won the tender, they could infer whether the other player outbid them or not. We note that theoretically, both competing players can be corrupt, yet for the sake of reducing complexity in the first implementation of the corruption game, we only introduced a corrupt option for the participant.

In order to translate this basic structure to a real-life scenario, we ask participants to take the role of a CEO of a construction company. In this game, the Ministry of Public Affairs advertises a big bridge building contract. Two companies are competing for this job by making bids over five rounds from the company’s budget (400.000 game-dollars). The best tender, i.e. the highest bid wins the entire bridge building contract (worth 120.000 $ each round). Equal bids lead to a split of the contract (60.000$ each). In order to ensure that participants understand the bidding structure of the game, we illustrate the structure with several examples and ask five test questions. When answering a question wrongly, participants had a second chance to answer the question correctly (across all three studies, participants answered more than 72% of the questions correctly on the first trial).

**Fig 2:**
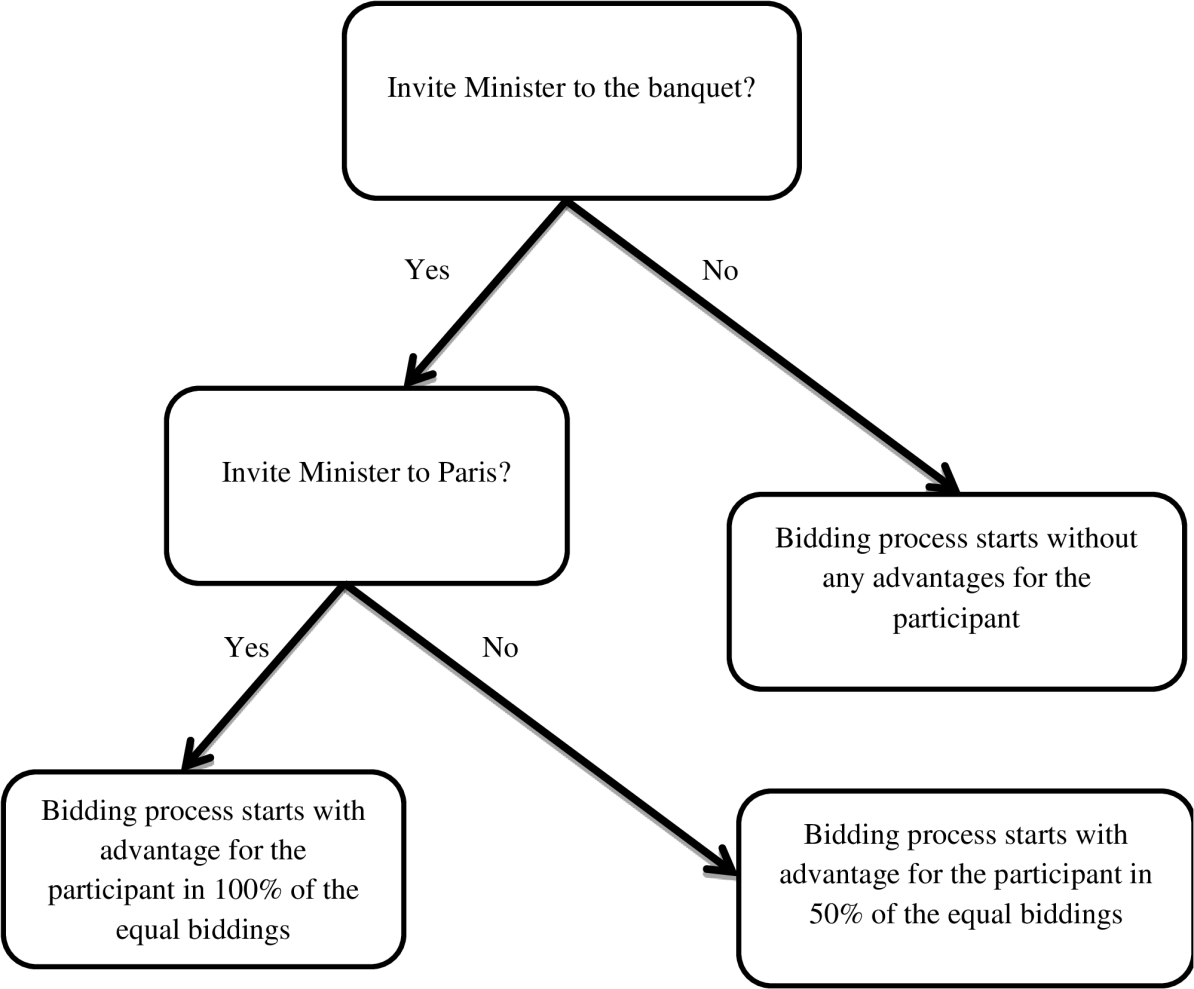
Game tree of the corruption game used in Study 1. Participants make step-wise decision about whether to invite the Minister or not.

The participants then face the decision whether or not to bribe the Minister. This criterion variable of corruption consisted of two levels in order to model a step-wise engagement in corruption (for an illustration of this decision structure see the game-tree in Fig 2). In a first instance, participants decide whether to invite the Minister to a company banquet, which ensures a bidding advantage in 50% of the equal biddings. This process is common in business transactions yet could be considered corruption as it ensures private benefits to the Minister and leads to a bidding advantage for the player [37]. Due to its common practice and legality (e.g. lobbyist practices), we refer to this choice as ‘ambiguous corruption’ in this manuscript.

For those who invite the Minister to the banquet a second invitation opportunity emerges which consists of an invitation of the Minister to a private vacation from the company’s budget. This invitation ensures advantages in 100% of the equal biddings. This second decision reflects a more severe and unequivocal act of corruption as the company’s budget is used to ensure private benefits for the Minister in return for full advantages in the allocation of the bridge building project. Due to its illegal character, we label this choice ‘severe corruption’.

## Results

The primary goal of the present study was to test the theorized positive link between perceived frequency and actual engagement in a specific corrupt behavior (see Table 2 for an overview of the frequency of corruption). For that purpose, we conducted two binary logistic regressions. In the first regression, we used the a priori corruption item as a predictor and the decision to invite the Minister to the banquet (no invitation vs. invitation) as a dependent variable. Results reveal that the perceived frequency of the corrupt act significantly influences the decision to invite the Minister to the banquet (*B* = 0.77, *Wald* = 5.08, *Exp(B)* = 1.86, *p* = .024). An increase in the perceived frequency of corruption of one standard deviation increased the odds of inviting the minister to the banquet by a factor of 1.86.

**Table 2:** Overview of the participants decisions in Study 1.

	Did participants invite the Minister?	
	Yes	No
First decision (invitation to banquet)	42	24
Second decision (invitation to vacation)	22	20

*Note*. The table illustrates the number of participants choosing to invite or abstain from invitation in both occasions. Note that only participants who invited the Minister to the banquet faced the second decision of whether to invite the Minister to the vacation

In the second binary logistic regression, we used the same predictor and used the decision to the vacation as a dependent variable (no invitation at all vs. invitation to vacation). Again, we find a significant effect (*B* = 0.64, *Wald* = 3.85, *Exp(B)* = 1.89, *p* < .05). An increase in the perceived frequency of corruption of one standard deviation increased odds of inviting the minister to the vacation by a factor of 1.89. For both types of corruption, the more frequent the participants perceived corruption to be, the more likely they engaged in it. Importantly, the work place norm scale did not significantly predict any of the dependent variables (all *ps* >.236).

## Discussion

The results confirm our hypothesis and show that the perceived norms about a specific corrupt behavior are associated with corrupt behavior. If participants perceived that inviting a Minister to a private vacation from company’s budget to obtain business advantages is relatively common, then they were also more likely to engage in this form of corruption themselves. Additionally, these perceived norms also predicted the likelihood of engaging in less severe and more ambiguous forms of corruption–inviting the Minister to the banquet. This is likely due to the strong similarity between the two types of invitations. The fact that only the corruption specific item–and not the entire work place norm scale–predicts corrupt behavior again underlines the context specificity of corruption.

To acknowledge an alternative explanation for the results, the possibility exists that the mere answering of a question about beliefs might have increased the salience of norms [38]. Participants might have acted more in accordance to their reported norms than they would have done otherwise. To exclude this possibility, we conducted a second study. This time we assessed perceived norms after the corruption game so that the assessment of corruption norms does not affect the decision to engage in corrupt behavior.

## Study 2

### Materials and Methods

**Fig 3:**
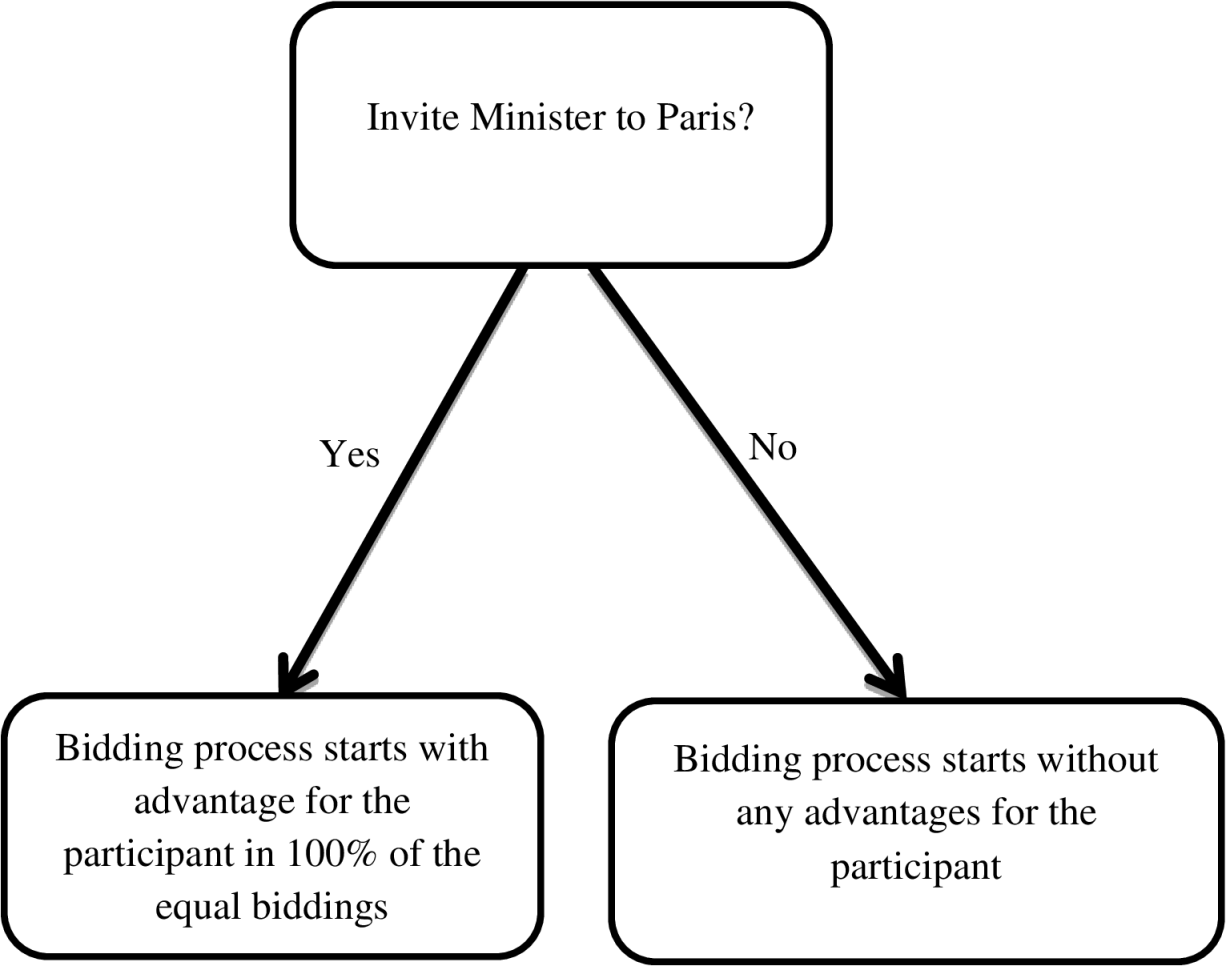
Game tree of the simplified corruption game. Participants directly face the decision whether to invite the Minister to the vacation.

In the second study, we also simplified the corruption game by removing the two-stepped structure of the dependent variable. We used such a step-wise structure of corruption to model many real-life occurrences of corruption that follow a slippery slope process [14]. Consequently, only those participants who engaged in ambiguous corruption faced the decision whether or not to engage in more severe corruption. This decision structure excluded a considerable proportion of the sample from making the second corruption decision. In order to distil the interpretability of the findings and to show the relationship between perceived norms and more severe forms corruption more clearly, we excluded the ambiguous corruption option. Participants therefore directly faced the choice whether they wanted to invite the Minister to the private vacation (i.e., more severe corruption) yielding advantages in 100% of the bidding rounds (see Fig 3 for a game tree of the simplified corruption game).

#### Participants and protocol

Students from the VU University Amsterdam (*N* = 119, *M*_*age*_ = 21.57, *SD*_*age*_ = 2.80, 63% = female) participated for course credit or money (2€). Participants first played the simplified corruption game and afterwards indicated their perceived descriptive norms. Apart from the simplification of the dependent variables, the game was administered in the identical way as in Study 1.

### Study design and tasks

#### Post hoc norms

After completion of the corruption game all participants answered one question which assessed the perceived frequency of the invitation to the vacation (i.e. ‘How many people do you think chose to invite the minister to the vacation’) to which answers are given on a 6 point scale ranging from ‘1’ (= nobody) to ‘6’ (= everybody). In between those end points of the scale participants could choose four percentiles of frequency (1–25%; 26–50%; 51–75%; 75–99%). We also manipulated public awareness with three conditions. The participants were either in a cubicle that had a webcam switched on, a webcam switched off or no webcam at all. This manipulation had no effect on any of the reported results.

## Results

In Study 2, we tested whether descriptive norms (measured after the corruption game) were associated with corrupt behavior. We calculated a binary logistic regression with the perceived norms as a predictor and the corrupt decision (i.e. invitation to vacation) as the dependent variable. We found a significant effect (*B* = 1.42, *Wald* = 26.72, *Exp(B)* = 4.162, *p* < .001). An increase of the perceived corruption norms by one standard deviation increased the odds of inviting the Minister to the vacation by a factor of 4.16.

Given the importance of priming effects [39] we also checked whether filling in the WPN scale in Study 1 triggered unethical behavior. We tested whether the level of corruption was higher in Study 1 compared to Study 2, in which participants did not fill in the WPN scale prior to the corruption game. We find no difference between the invitation to the vacation (χ² = .119, *p* = .730), nor when comparing ambiguous corruption in Study 1 with severe corruption in Study 2, hence the first choices in both studies, (χ² = 1.16, *p* = .28).

## Discussion

The results again show a strong link between corrupt behavior and the descriptive norms about this form of corruption. In Study 2, participants were asked for their norms perception after they played the corruption game. It is possible that participants changed their norms perception according to their behavior in the game (i.e. norms serving as a rationalization). Taken together with Study 1, in which norms were assessed before the corruption game, we find strong support for the close link between descriptive norms and corrupt behavior in the game. The more frequent participants perceived the corrupt behavior to be, the more likely they were to behave corruptly in the corruption game themselves.

## Study 3

One way to interpret the obtained post hoc norm differences in Study 2 are rationalization strategies. People might rationalize and justify corrupt behavior by indicating that it is ‘a common thing to do’. Previous research investigating unethical behavior supports this notion [34,40] by showing that people cheat and lie more if they have excuses or explanations at hand. Descriptive norms might function as such an excuse [41] and a normalization process of corruption might come into motion [31]. That is, people might adjust their own perceived norms to their own behavior. We argue that besides functioning as a rationalization of corrupt behavior, descriptive norms also provide an a priori benchmark for corrupt behavior.

In order to investigate this assumption and to provide causal evidence, we set up a third study in which we tested whether a manipulation of descriptive norms can influence corrupt behavior. Previous research indicates that small morality related primes can reduce unethical behavior such as cheating [40,42]. Using descriptive norms related primes has been shown to impact a wide array of behavior ranging from an increase in tax compliance [43] to enhanced energy saving behavior [44,45]. However, an empirical test of the impact of descriptive norm primes on corruption is lacking. To investigate the causal link from perceived norms to corrupt behavior we set up an experiment in which we manipulated descriptive norms by presenting short primes to participants prior to the corruption decision.

### Materials and Methods

We used the same simplified study design as in Study 2. Additionally, we reduced the rounds of bidding from five to one in order to reduce the complexity of utility calculation for the participants. Previously the participants had to anticipate the advantage of corruption for five rounds of bidding, now they merely had to anticipate the advantage of corruption for one round of bidding. In economic terms, the benefit of corruption remained the same yet the calculation of the benefits of corruption was easier for the participants.

#### Participants and protocol

We conducted an online study (*N* = 259; *M*_*age*_ = 35.65; *SD*_*age*_ = 11.54; 42.1% = female) in English via Amazon Mechanical Turk. Participants needed to reside in the United States, and have more than 5000 approved HIT with an approval rate of at least 98%. Participants were reimbursed with 1$ for their participation. Participants first read the instructions to the corruption game. Before making the decision about whether or not to invite the Minister to the vacation, they received one of three norm statements. In the anti-corruption norms condition we presented the participants with a prompt stating ‘Almost nobody invites the Minister’. In the pro-norm condition the prompt read ‘Almost everybody invites the Minister‘. In the control condition, participant received no such prompt. After completion of the corruption game, we again assessed the perceived descriptive norms with the same question as in Study 2. In addition to that, half of the participants received a time pressure prompt, which did not affect the reported results.

## Results

### Measurement of perceived descriptive norms

In order to check whether the norm manipulation did indeed influence the perceived norms of corruption, we conducted an ANOVA with the norm manipulation (three levels) as a predictor and the perceived norms item (assessed after the game) as the dependent variable. The results reveal significant differences in perceived norms between all three groups (*R²* = .34, *F* (2,257) = 70.91, *p* < .001). Participants in the in the anti-corruption norms condition perceived corruption to be least common (*M* = 2.57, *SD* = 1.33), compared to participants in the control condition (*M* = 4.03, *SD* = 1.25) who in turn perceived corruption to be less common than participants in the pro-norm condition did (*M* = 4.74, *SD* = 1.06). All post-hoc group-wise comparisons are significant (all *ps* < .002; Bonferroni corrected).

### Hypotheses testing

First off, in comparison to the other two studies, we find that the overall corruption level was higher in the online study compared to the previous two lab studies (χ² = 9.37, *p* = .009), most likely to due to the increased anonymity of the internet. We then tested whether the manipulation of norms significantly affected the decision to engage in corruption by calculating logistic regression analyses with the norm manipulation as a predictor variable and the decision to engage in corruption as a dependent variable. In this third study, participants in the anti-corruption norms condition were significantly less likely to engage in corruption than participants in the control condition (*B* = 0.83, *Wald* = 6.43, *p* = .011, *Exp(B)* = 2.30). The odds of engaging in corruption were 2.3 times lower in the anti-corruption norm condition than in the control condition.

In addition, we find a significant effect between the anti-corruption norms condition and in the pro-corruption norms condition (*B* = 0.79, *Wald* = 5.87, *p* = .015, *Exp(B)* = 2.30). The odds of engaging in corruption were 2.3 times higher in the pro-corruption norms condition compared to the anti-corruption norms condition. No significant difference between the control and pro-norm condition existed (*p* = .89). Hence, the anti-corruption norm prime significantly reduced the level of corrupt behavior in the game compared to the control and the pro-corruption norm condition.

We additionally tested whether perceived norms mediate the effects of manipulated norms. Two mediation analysis using bootstrap analyses for the two significant effects (anti vs. control; anti vs. pro norms) indicate full mediation in both cases (anti vs. control: CI95% [-1.83; -0.73]; anti vs. pro norms: CI95% [-2.59; -1.2]).

## Discussion

The results of the third study illustrate that descriptive norm prompts can influence subsequent levels of corrupt behavior. When participants received a short prompt indicating a low frequency of corrupt behavior the level of corruption decreased drastically in comparison to the control and pro-corruption norms condition. Interestingly, the results suggested no difference in corrupt behavior between the pro-corruption norm condition and the control condition, which indicates that the respective corrupt behavior was generally perceived to be common.

## General Discussion

The impact of descriptive norms on corrupt behavior has been frequently theorized but–as far as we know–never experimentally tested [17,29]. The present set of studies provides first empirical support for the assumed link. Perceived descriptive norms were associated with the subsequent corrupt behavior (Study1). In order to rule out that increased salience of norms caused this effect, we showed that the perceived norm differences also exist when assessing norms after the behavioral measure of corruption (Study 2). Finally, short statements containing descriptive norm information successfully influenced the likelihood of making corrupt decisions. Specifically, information that indicates a low frequency of corrupt behavior (anti-corruption norms) reduced the level of the ensuing corrupt behavior (Study 3). Anti-corruption norms likely drove the effect because the perceived frequency of corruption in the sample was relatively high.

People rely on descriptive norms as a guideline to make corrupt decisions, especially in situations in which they have little or no own experience [21,46]. We put participants in such a novel and uncertain situation in which descriptive norms are primarily based on beliefs and not on own experience. The fact that participants heavily relied on norms indicates that people who encounter such novel situations might be especially prone to rely on descriptive norms as decision benchmarks. Think of newcomers in organizations who do not have their own experiences about the business practices: if these newcomers apprehend that corruption is not commonplace they likely abstain from it as well. Our results sketch a new tentative path how norm related reminders might shape low corruption norms. Small reminders and prompts could potentially provide a ‘nudge’ [47] to reduce corruption–especially in contexts in which people do not have first-hand experience and/or falsely believe that a high proportion engages in corruption.

From an empirical perspective, the corruption game provides a novel experimental tool that allows researchers to look at the psychological aspects involved in corruption. Furthermore, it allows testing corrupt decisions in a context in which participants have little own first-hand experience, even though it represents a typical corruption dilemma. By masking corruption in a bidding game, it allows to empirically study corruption while avoiding social desirability effects. Using the novel corruption game, we provide a first illustration of the link between perceived descriptive norms and corruption.

It is noteworthy that neither monetary incentives nor punishment existed in the three studies presented in this manuscript. Considerations regarding material outcomes, or cost-benefits calculations unlikely account for the present findings. Contrary to most corruption research in which reward and punishment play vital roles, we opted for this design as it enabled us to identify descriptive norms as a prime candidate for uncovering the complexity of corruption in an isolated environment. Indeed, one important issue for future research lies in the examination of how normative influences work across a variety of contexts, including those where incentives, and punishment, are bound to affect corruption. Future research could also look at whether descriptive norms influence the perceived punishment of corruption. Further, does the perceived frequency of corruption increase when the potential gain is high? While the link between frequency of punishment and descriptive norms might seem plausible, how is the relationship between severity of punishment and descriptive norms? This question is especially interesting in moral gray areas like in the ambiguous corruption presented in Study 1.

In Study 3, we show that norms are subject to external influences as short prompts successfully influenced perceived descriptive norms. On a positive note, we found that especially anti-corruption norm prompts effectively reduce the level of subsequent corrupt behavior. Since we used a rather explicit manipulation of norms in Study 3, using more implicit manipulations is another interesting avenue for corruption research, especially given that many forms of abuse of power happen without conscious awareness of it [13].

The present set of studies shows that the belief about the frequency of corruption influences the likelihood of engaging in corruption. The fact that we asked participants to imagine a situation of corruption rather than actually placing them in a potentially corrupt situation limits the generalizability of the findings. Yet given the illegal character of corruption, studying corruption in its real-life context poses a major challenge for corruption research. Corruption games, such as the one presented here provide a way to study corruption experimentally. Future studies could increase the generalizability of the obtained results by additionally including economic factors such as rewards and punishment, testing the impact of descriptive norms on other forms of corrupt behavior [16] and using more diverse samples [48].

Additional studies could also explore how corruption norms are shaped. For example, how does news coverage of corruption in the media impact corrupt behavior? Previous research suggests that media coverage thoroughly impacts descriptive norms which in turn lead to imitation of highly publicized behavior [49,50]. Whether a similar ‘copycat’ corrupt behavior follows highly publicized cases of corruption (e.g. the Madoff case), could be a fascinating topic for future research.

## Conclusion

We provide first empirical support for the importance of the more subtle psychological factors of perceived descriptive norms on corrupt behavior. Perceiving that corruption is widespread crucially influences the decision to engage in corrupt behavior–a perception that can be influenced with small norm prompts. Due to the importance of descriptive norms for daily decisions, Elster referred to social norms as the ‘cement of the society’ [51]. In highly corrupt social contexts the ‘cement of social norms’ stabilizes corruption while in low corruption context it does the opposite: enabling non-corruption to be the ‘normal thing to do’. To come back to the initial example, the current set of studies suggest that your answer to the question whether you would invite the Minister depends on your beliefs about this corrupt behavior of others. Believing that nobody invites the Minister lowers the chances of acting corruptly while perceiving that such an invitation reflects a common business practice increases the chances of you doing likewise, thinking: “I invite the Minister, who doesn’t?”.
